# Carbamate Pesticides: Shedding Light on Their Impact on the Male Reproductive System

**DOI:** 10.3390/ijms23158206

**Published:** 2022-07-26

**Authors:** Sílvia Moreira, Ricardo Silva, David F. Carrageta, Marco G. Alves, Vicente Seco-Rovira, Pedro F. Oliveira, Maria de Lourdes Pereira

**Affiliations:** 1 Department of Medical Sciences, University of Aveiro, 3810-193 Aveiro, Portugal; s.moreira@ua.pt; 2 CICECO-Aveiro Institute of Materials, University of Aveiro, 3810-193 Aveiro, Portugal; 3 LAQV-REQUIMTE and Department of Chemistry, University of Aveiro, 3810-193 Aveiro, Portugal; rsilva11@ua.pt; 4 Multidisciplinary Unit of Biomedicine Investigation (UMIB), Department of Anatomy, Abel Salazar Institute of Biomedical Sciences (ICBAS), University of Porto, 4050-313 Porto, Portugal; davidcarrageta@gmail.com (D.F.C.); alvesmarc@gmail.com (M.G.A.); 5 Laboratory for Integrative and Translational Research in Population Health (ITR), University of Porto, 4050-600 Porto, Portugal; 6 Biotechnology of Animal and Human Reproduction (TechnoSperm), Institute of Food and Agricultural Technology, University of Girona, 17003 Girona, Spain; 7 Unit of Cell Biology, Department of Biology, Faculty of Sciences, University of Girona, 17003 Girona, Spain; 8 Department of Cell Biology and Histology, University of Murcia, 30100 Murcia, Spain; vicente.seco@gmail.com

**Keywords:** carbamates, male fertility, endocrine disruptors, endocrine system, acetylcholine, NR0B2 (Nuclear Receptor Subfamily 0 Group B Member 2), kisspeptin

## Abstract

Carbamates are widely used and known around the world as pesticides in spite of also having medical applications. This class of chemicals is classified as acetylcholinesterase inhibitors, blocking acetylcholine hydrolyzation in a reversible manner. Their lack of species selectivity and their reported high toxicity can induce, upon exposure, adverse outcomes in male fertility that may lead to infertility. In addition, they are also considered endocrine-disrupting chemicals and can interfere with the hypothalamic–pituitary–testicular axis, essential for the normal function of the male reproductive system, thus being able to provoke male reproductive dysfunctions. Although the molecular mechanisms are not fully understood, various signaling pathways, such as those mediated by acetylcholine or kisspeptin, are affected by exposure to carbamates, thus compromising steroidogenesis and spermatogenesis. Over the last decades, several studies, both in vitro and in vivo, have reported a myriad of negative effects of carbamates on the male reproductive system. In this review, an up-to-date overview of the impact of carbamates on the male reproductive system is discussed, with an emphasis on the role of these compounds on acetylcholine regulation and the male endocrine system.

## 1. Introduction

Carbamates are mostly used as pesticides worldwide, despite their interesting medical applications, such as in the treatment of myasthenia gravis, Alzheimer’s disease, or glaucoma, among others [1]. Carbamates were firstly extracted from a West African plant, Calabar bean, *Physostigma venenosum*, and are classified as esters of N-methyl carbamic acid [2]. This class of chemicals is known to lack residue persistency in the environment and in mammalian species due to their rapid hydrolyzation to, usually, less toxic metabolites, amine, and carbon dioxide (CO_2_), which are easily excreted from the organism [3]. However, because of their lack of species selectivity, and their lipophilicity and toxicity, its overexposure and/or chronic exposure poses a serious threat to the environment, to human beings and to other animal species [4,5]. Compared to other classes of pesticides, carbamates exhibit one of the widest ranges of subtypes (carbendazim, carbaryl, carbofuran, aminocarb, thiodicarb, mancozeb, among others). Each compound has its application (e.g., insecticide, fungicide, and herbicide), characteristics, and harmful effects (as reviewed by King and Aaron [1]). Despite these differences, the compounds from the carbamate´s family share the common trait of being both acetylcholinesterase (AChE) inhibitors, through its carbamylation, and endocrine-disrupting chemicals (EDCs), interfering with the hypothalamic–pituitary–testicular (HPT) axis, which may cause several reproductive problems [4,6,7,8].

The endocrine system consists of a series of glands throughout the body that produce and secrete hormones to regulate a wide range of physiological processes, such as respiration, metabolism, reproduction, sensory perception, movement, sexual development, and growth. The main hormone-producing glands are the hypothalamus, pituitary, parathyroid, pancreas, thyroid, adrenal, pineal, ovaries, and testis. Each gland produces and is under the influence of specific hormones or hormonal cues, controlling the body homeostasis. There are several factors that can influence the function of endocrine organs, including aging, certain diseases and conditions, stress, environment, and genetics (7). EDCs are compounds that can mimic the action of hormones, binding to their receptors and inhibiting or stimulating the respective cascade and the HPT axis. In addition, EDCs can also affect the concentration of hormones through the stimulation or inhibition of their degradation, availability, or synthesis [9].

Carbamates are known EDCs and their exposure has been linked to male infertility. In this review, we provide a summary of the literature examining the formulations and possible mechanisms of the action underlying the effects of carbamate pesticides on male fertility. In order to do that, the search was conducted on 8 March 2022 using SCOPUS search. An advanced search was performed using the formula: (TITLE–ABS–KEY (carbamates AND male fertility) or TITLE–ABS–KEY (carbamates AND endocrine disruptors)). This search retrieved 109 results. All resulting articles were manually screened. From the initial manuscripts retrieved by the keyword search, 30 were excluded because they were not original research papers and 8 for not being written in the English language. From the remaining 71 manuscripts we further excluded the studies that were not conducted on human beings or vertebrates, and 1 that could not be retrieved. The final selection of 59 manuscripts further included relevant papers addressing important introductory concepts.

## 2. The Role of Acetylcholine in Male Reproductive Function

**Figure 1:**
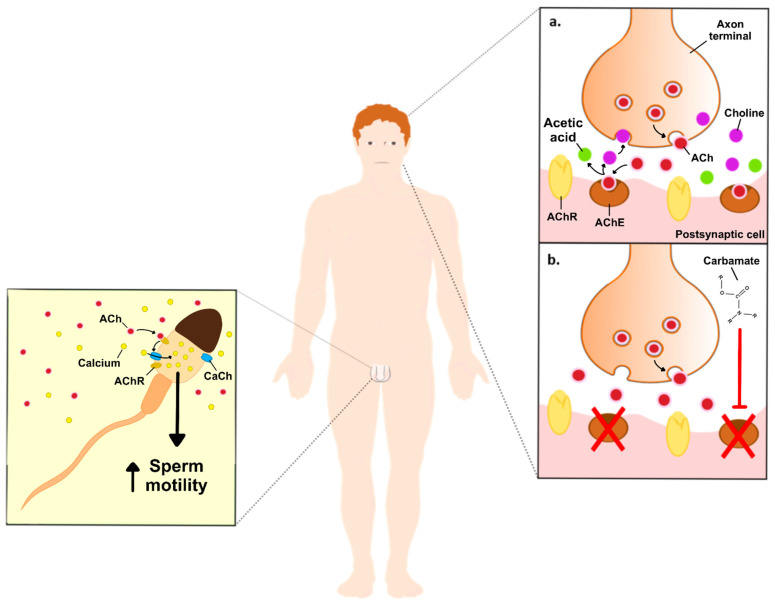
Schematic illustration of the effects of carbamates on neuronal ACh, and the hypothetical effects on non-neuronal ACh in the male reproductive system. **Image right side:** (**a**). ACh vesicles (red dots) are released to the synaptic clef, where they will bind to its receptors, while the excess is converted into acetic acid (green dots) and choline (purple dots), by the AChE. Choline is then taken up by the neuron to be resynthesized into new ACh. (**b**). Inhibition of AChE, upon exposure to carbamates, increases the levels of ACh on the synaptic cleft, thus producing its toxic effects. **Image left side:** ACh increases sperm motility through the stimulation of Ca^2+^ influx into the spermatozoa. Abbreviations: ACh—acetylcholine; AChE—acetylcholinesterase enzyme; AChR—acetylcholine receptor; CaCh-Ca^2+^ channels.

The toxicity of carbamates occurs due the inhibition of the AChE through carbamylation, which stops the degradation of the neurotransmitter acetylcholine (ACh) and leads to its accumulation in brain synapses and neuromuscular junctions. At physiological conditions, synaptic AChE prevents the accumulation of ACh and consequent overstimulation of the ACh receptor through its hydrolyzation to acetic acid and choline. Next, acetic acid feeds into the Krebs cycle, while choline is taken up by the neuron and resynthesized into new ACh. In this way, AChE inhibition results in the accumulation of ACh, overstimulating the muscarinic and nicotinic ACh receptors (Figure 1). The accumulation of ACh affects the whole organism and the most observed symptoms include sweating, excessive salivation, constricted pupils, blurred vision, lacrimation, wheezing, gastrointestinal issues including nausea, vomiting, and diarrhea, urinary incontinence and, in more severe cases, paralysis, cyanosis, and respiratory issues, which can last from a few hours to a few days or weeks [1]. Contrarily to organophosphates, another class of anticholinesterase pesticides, carbamates reversibly inhibit AChE, and are, therefore, less toxic [4].

Biomarkers of toxicity of anti-AChE pesticides, like carbamates, were found to reduce epididymal and testicular sperm counts, as well as decrease serum testosterone concentration [10]. Many of the actions of ACh, including on the male reproductive system, are induced by its effects on muscarinic ACh receptors (mAChR) or nicotinic ACh receptors (nAChRs). The binding of acetylcholine to its receptors promotes Ca^2+^ influx, which activates Ca^2+^-dependent pathways including stimulation of the adenylyl cyclase activity and increased cyclic AMP (cAMP) levels, activation of the phosphoinositide 3-kinase (PI3K) pathway, and Ca^2+^/calmodulin-dependent protein kinase (CaMK)-dependent pathways [11,12]. The mAChR are located throughout the human organism, mainly at organs innervated by the parasympathetic system with particular emphasis on exocrine glands, including sweat and salivary glands, but also the heart, gastrointestinal tract, eye, and testis [13,14,15]. These include five distinct subtypes (M_1_-M_5_), and their activation is known to be involved in cell proliferation, differentiation, growth, among other functions. M_1_, M_3_ and M_5_ mAChRs preferentially couple to G-proteins of the G_q/11_ family, while M_2_ and M_4_ mAChRs preferentially activate G_i/0_-type G-proteins [16,17,18]. Han et al. studied the knockdown of M_1_, M_3_ and M_5_ and found that the M_5_ knockdown disrupted spermatogenesis and affected the expression of proteins in the blood-testis barrier (BTB) and ectoplasmic specializations. Furthermore, the M_5_ knockdown decreased the expression of pleckstrin homology-like domain, family B, member 2 (Phldb2), both in germ cells and Sertoli cells [15]. Phldb2 is a PH domain-containing protein that is highly sensitive to phosphatidylinositol 3,4,5-triphosphate (PIP3) and phosphatidylinositol 4,5-biphosphate (PIP2). Among its functions, Phldb2 has an important role in ACh receptor aggregation in the postsynaptic membrane, thus being a key component of synaptic podosomes, which are involved in the extracellular matrix remodeling [19]. Phldb2 also mediates focal adhesion disassembly as well as cell polarization and migration. Thus, the authors could conclude that the M_5_ knockdown decreased the expression of Phldb2 and disrupted the BTB and ectoplasmic specializations, impairing spermatogenesis [15].

Beyond mAChRs, ACh can also stimulate an unknown variety of ionotropic nAChRs with subtype-specific arrangements [20]. In more detail, these receptors belong to the super-family of homologous Cys-loop ion channel receptors, which consist of nine α and three β subunits. Both homomeric or heteromeric assembly of five subunits generate several distinct subtypes, which share a common basic structure but have specific pharmacological and functional properties [21]. Similar to mAChRs, the absence of nAChRs or the presence of reduced levels of ACh leads to reduced sperm motility, thus impairing male fertility [22]. Although the mechanisms remain to be elucidated, Bray et al. hypothesized that the nAChRs play a role on Ca^2+^ influx, which is essential for sperm motility and hyperactivation [22]. On the other hand, Arıcan et al. treated male Sprague–Dawley rats with acetamiprid (12.5, 25, and 35 mg/kg/day), a selective agonist of nAChRs, for 90 days and observed a decreased sperm count and increased apoptotic index in the testis upon exposure to the highest concentration. In addition, increased oxidative stress markers were reported, which were highlighted as the likely cause for the observed detrimental effects [23]. Taken together, these results suggest that nAChRs take part in a finely regulated pathway that modulates the testicular function, but further studies are needed to unveil the involved molecular mechanisms.

ACh was also found, along with crucial components of the cholinergic system, in mammalian cells not innervated by neurons, i.e., non-neuronal ACh and non-neuronal cholinergic system, respectively [24]. Namely, non-neuronal ACh was detected in the sperm of rabbit, bull, and man [25,26]. Moreover, there is evidence that the non-neuronal cholinergic system is widely expressed within the reproductive tract of male rats [27]. Via auto- and paracrine modes of action, and as previously mentioned for neuronal ACh, non-neuronal ACh also promotes cell proliferation and differentiation, as well as regulation of cell-cell contact, locomotion, and transport of ions and water [24,25,26,27,28]. Previous studies reported that interfering with the ACh cascade affects male reproductive potential. Sliwa observed that a high concentration of ACh (5 mg/mL) reduced in vitro mouse spermatozoa migration [29]. Sastry et al. reported that 1 μM of 2-benzoylethyltrimethylammonium (BETA), a choline acetyltransferase inhibitor, rapidly decreased human sperm motility (80% after 5 min treatment). In addition, a decrease in sperm motility by 95% was observed after 1 h treatment. Interestingly, these authors hypothesized that ACh is potentially synthesized by human sperm, exhibiting an autocrine and/or paracrine role [30]. Likewise, interferences in the non-neuronal cholinergic system caused by pharmacological substances like atropine, an anticholinergic drug that acts as a competitive yet reversible mAChR antagonist, were demonstrated to lead to impaired fertility, due to the inhibition of the contraction of the seminal vesicles during the process of emission. Sato et al. reported that male Sprague–Dawley rats treated with 125 mg/kg/day of atropine for 10–17 days had increased concentration of sperm in the *vas deferens* and observed a lower pregnancy rate when mated with females, as compared to the control group. These results led the authors to suggest that the inhibition of mAChRs by atropine led to the impairment of sperm and semen transport [31]. In a previous study, Ban et al. already had reported that Sprague–Dawley male rats treated with 125 mg/kg/day of atropine for 1 week led to a lower pregnancy rate. In addition, these authors also studied the effects of another mAChR antagonist, (2R)-N-[1-(6-aminopyridin-2-ylmethyl)piperidin-4-yl]-2-[(1R)-3,3-difluorocyclopentyl]-2-hydroxy-2-phenylacetamide, and reported that the treatment of 100 mg/kg/day for 4 weeks also lowered the pregnancy rates. Interestingly, no detrimental effects were observed after a 1-week withdrawal, suggesting that the effects of mAChR inhibition conferred by this compound are reversible [32]. More recently, it was reported in rats that the contraction of the testicular capsule, a complex fibrous structure consisting of various and distinct layers of tissues, like tunica albuginea, is associated with M_3_ receptors. Upon induction with cholinergic agonists, the activation of M_3_ receptors leads to Ca^2+^ influx through L-subtype and Ni^2+^ sensitive voltage-dependent Ca^2+^ channels, Ca^2+^ release from sarcoplasmic reticulum, as well as regulation of Ca^2+^ levels by the mitochondria. As such, and as explained by the authors, the contraction of testicular capsule has a functional involvement in sperm transport from the testes to epididymis. In this way, ACh present in non-neuronal sites may induce or modulate their contractions, allowing the correct movement of sperm from the testes [33]. In accordance, Nieto-Cerón et al. showed that AChE may play an important role in the male reproductive physiology, as they found significant levels in the human prostate [34]. Nevertheless, it remains to be studied whether the reduced cholinesterases (ChE) activities observed in semen are a cause or consequence of infertility, as the authors proposed [34]. Taken together, it is possible to conclude that ACh plays a regulatory role on male fertility, especially on spermatozoa, but further studies are needed to disclose the involved molecular mechanisms.

Carbamates can interfere within the normal neuronal ACh cascade, which means that, theoretically, they will also play a role in the regulation of non-neuronal ACh levels in the male reproductive system, since the non-neuronal cholinergic system is part of it. The direct effects of carbamates exposure to the testicular function, spermatogenesis, and sperm function, however, remains to be disclosed. Radhakrishnan et al. reported that elevated concentrations of ACh induced oxidative stress in the testis, thus diminishing sperm motility [35]. This outcome was found, however, to be reverted by a mild intramuscular dose of Botulinum toxin in the inferior spermatic nerve plexus, a place known to regulate steroidogenesis [35]. The Botulinum toxin acts through the cleavage of the SNARE proteins, preventing the release of ACh vesicles [36]. These data suggest that the carbamates-induced accumulation of ACh could lead to similar results, although further studies are needed to confirm this hypothesis.

## 3. Carbamates as Endocrine-Disrupting Chemicals (EDCs)

Carbamate pesticides have endocrine-disrupting properties that include disruption of the male reproductive system through the perturbation of the HPT axis, although the molecular mechanisms are not fully understood [4]. As the name indicates, this axis is composed by the hypothalamus, pituitary gland, and testes, and its normal function is crucial to provide the right amounts of hormones for male sexual development and function, and so any abnormality can lead to male infertility [37].

**Figure 2:**
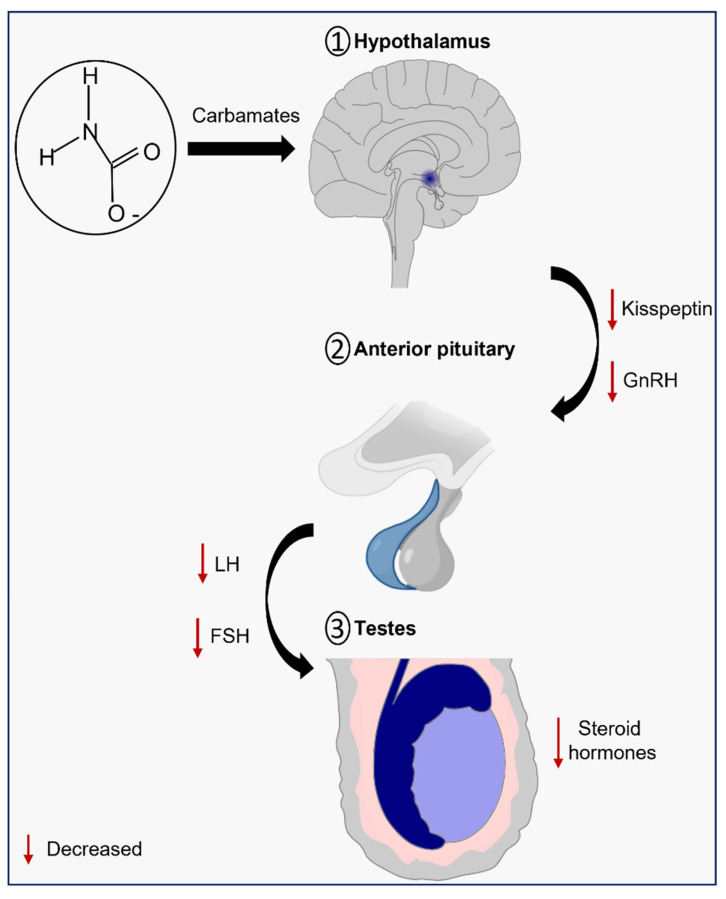
Schematic illustration of the effects of carbamates on the hypothalamic-pituitary-testicular (HPT) axis. Exposure to carbamates leads to inhibition of kisspeptin neurons and, consequently, to low levels of GnRH in the hypothalamus. In this sense, the synthesis of LH and FSH in the anterior pituitary is arrested. Finally, the synthesis of steroid hormones in the testis is compromised, leading to male fertility problems. Abbreviations: GnRH—gonadotropin-releasing hormone; LH—luteinizing hormone; FSH—follicle stimulating hormone.

The hypothalamus is responsible for the secretion of pulsatile gonadotropin-releasing hormone (GnRH), which stimulates the synthesis and secretion of the gonadotropins luteinizing (LH) and follicle-stimulating (FSH), by the adenohypophysis. Then, FSH travels through the bloodstream and binds to its receptors on Sertoli cells, while the LH binds to the LH/chorionic gonadotropin receptor (LH/CG-R) present on the membrane of Leydig cells and stimulates steroidogenesis (Figure 2) [38,39]. The act of mobilizing cholesterol into mitochondria is the first and limiting step in the multi-phase process of testosterone production [40]. Cholesterol mobilization is mediated by the action of the translocator protein (TSPO) and the steroidogenic acute regulatory protein (StAR), both dependent on LH-induced increase on cAMP levels and cAMP-dependent phosphorylation by protein kinase A (PKA). Then, the cytochrome P450 enzyme (CYP11A1), which is present on the matrix side of the inner mitochondrial membrane, catalyzes the conversion of cholesterol into pregnenolone. Three enzymes—3β-hydroxysteroid dehydrogenase (HSD3B), 17α-hydroxylase/17,20 lyase (CYP17A1), and type 3 17β-hydroxysteroid dehydrogenase (HSD17B)—progressively convert pregnenolone into testosterone in the smooth endoplasmic reticulum [41]. Testosterone, then, regulates the pituitary’s production and secretion of LH through a negative feedback mechanism [42].

Several studies highlight the effects of carbamate exposure on the HPT axis. Recently, it was observed that rats exposed to carbendazim (a carbamate fungicide), to daily doses of 100 mg/kg by oral gavage for 8 weeks led to a significant decrease in the serum levels of sex hormones. Moreover, a decrease in serum levels of inhibin B, a glycoprotein hormone secreted by Sertoli cells that regulates the pituitary in a negative feedback mechanism, was observed. Contrarily, a significant increase in thyroid stimulating hormone (TSH) concentrations was noticed. All these changes, together with the inflammatory burst and the induction of oxidative stress, lead to toxic actions that negatively impacted spermatogenesis and steroidogenesis [43]. In line with the previous study, Elsharkawy et al. showed that male rabbits exposed to mancozeb (another carbamate fungicide), at 100 mg/kg administered orally twice per week for 12 weeks, suffered a considerable reduction in serum concentrations of testosterone and gonadotropins. Moreover, a significant decrease in testicular androgen-dependent enzymes, like lactate dehydrogenase (LDH), non-prostatic acid phosphatase (ACP) and alkaline phosphatase (ALP), was noted. All these modifications lead to reduced sperm viability and a high number of abnormal spermatozoa in the cauda epididymis. Histopathological evaluation of the testicular tissue showed disruption of the germinal epithelium as well as a reduction of spermatogenic cells. Interestingly, upregulation of the steroidogenic enzyme HSD3B has been reported despite the observed vacuolization of Leydig cells [44]. Another study, with mancozeb (0.14 mg administered through food, for 30 days), reported reduced weight, volume, and histopathological alterations on the testis of red avadavat (*Amandava amandava).* These authors also hypothesized that the disruption of neurohormones might contribute to neurotransmitter imbalance in hypothalamic neurons, thus leading to neuroendocrine dysregulation of reproductive development and maintenance [45]. Meng et al. reported that upon exposure to high concentrations (20 and 200 µg/L for 30 days in their living water) of methomyl, a carbamate insecticide, male tilapia (*Oreochromis niloticus*) showed increased expression of *gnrh2*, *gnrh3*, *erα*, and *erβ* genes in the hypothalamus, *gnrhr* and *fshβ* genes in the pituitary, along with *cyp19a*, *fshr*, and *erα* genes in the testes. Decreased levels of *lhr*, *star*, *hsd3b*, and *arα* genes in the testes, and the *lhβ* gene in the pituitary, were observed. All alterations of these gene levels led to reproductive dysfunction in male tilapia, which was found to be reversible at 20 µg/L but not at 200 µg/L. As such, these authors considered the 200 µg/L the threshold concentration for methomyl-induced irreversible endocrine disruption in male tilapia [46]. Guo et al. administrated ziram orally, a carbamate fungicide, to 35-day old Sprague–Dawley rats (2 or 4 mg/kg/day) for 4 weeks and observed a lower concentration of serum testosterone and FSH, and a decreased number of Leydig cells. In addition, several steroidogenic enzymes were also downregulated, including *Star*, *Cyp11a1*, *Cyp17a1*, *Hsd3b1*, *Hsd11b1*, and *Hsd17b3*. To further study the molecular mechanisms, these authors also established primary cultures of immature Leydig cells isolated from the same rats and treated them with ziram (0.5–50 μM). Data obtained from the in vitro study supported the results from the in vivo model, as a reduced androgen production due to downregulation of steroidogenic enzymes was observed. It was also reported that ziram induced Leydig cells´ apoptosis by upregulating BAX, a pro-apoptotic protein, and downregulating BCL2, an antiapoptotic protein [47]. Taken together, it is suggested that exposure to carbamates impairs steroidogenesis through the downregulation of steroidogenic enzymes and/or toxicity to Leydig cells. The molecular mechanisms, however, are still poorly understood, which highlights the importance for further studies in this field.

## 4. Carbamates and Kisspeptin

Kisspeptin, produced by kisspeptin neurons located in the hypothalamus, is a neuropeptide hormone responsible for stimulating the GnRH secretion, crucial for the normal function of the HPT axis [48,49]. More recently, the role of kisspeptin on the pathology of the HPT and its impact on human reproduction was highlighted [50]. Indeed, this signaling system is considered an essential regulatory part of the HPT axis, since its pulsatility, together with GnRH pulses, are essential for sexual maturation [51,52]. Interestingly, GnRH neuron-specific *Kiss1R* knockdown in mice (GkirKO) has been shown to lead to hypogonadotropic hypogonadism, i.e., lower concentrations of circulating gonadotrophins and testosterone. The knockout males exhibited a dramatic reduction in testicular size when compared to wild-type mice, as well as a defect in spermatogenesis, with the epididymis having fewer sperm present in the lumen. In this sense, the authors were able to conclude that disruption of the kisspeptin signaling solely in the GnRH neuron results in infertility, meaning that kisspeptin signaling in hypothalamic GnRH neurons plays a crucial role for the proper functioning of the reproductive HPT axis [53].

The kisspeptin family encompasses various peptides, all of which with the ability to bind to the KISS1 receptor (*Kiss1R*). Besides the brain, the kisspeptin system has been reported to also be expressed in the peripheral tissue, such as the gonads, adipose tissue, and liver, thus revealing a wide spectrum of actions in the body. Regarding the gonads, kisspeptin receptors are mainly expressed in the testis of non-mammalian and mammalian vertebrates, with specific differences related to age or species [54]. Additionally, kisspeptin expression has been reported in human spermatozoa, specifically at the post-acrosomal region of the sperm head, and fertile men show higher kisspeptin serum levels compared to infertile men [55]. Hsu et al. reported, for the first time, that kisspeptin and KISS1R are expressed both in mouse seminiferous tubules and interstitial cells. According to these authors, the expression of kisspeptin mainly occurs in Leydig cells, while KISS1R is expressed by germ cells, like spermatids and mature spermatozoa. Nevertheless, kisspeptin did not seem to affect steroidogenesis directly in Leydig cells, but instead induced testicular degeneration through GnRH. Furthermore, these authors also showed that KISS1R is specifically expressed in the acrosome region in spermatozoon, yet kisspeptin was found not to regulate the acrosome reaction, but to regulate the fertilizing capacity of spermatozoa during capacitation and may also protect spermatozoa function in the reproductive tracts [56]. In another study, *kiss2* mRNA was shown to have a similar expression pattern in the brain, pituitary, and gonads of both sexes of Nile tilapia (*Oreochromis niloticus*). Its expression in the gonads was significantly higher in the immature stage compared to the mature one, suggesting its involvement in the early gonadal maturation of the species. Additionally, in both immature sexes, an intraperitoneal injection of synthetic kisspeptin (Kp-10) increased the GnRH and gonadotropin (GTH) subunit genes in the brain and 17β-estradiol (E2) and 11-ketotestosterone (11-KT) levels in blood plasma. Kp-10 treatment in males was also associated with an acceleration of gonadal maturation [57]. Moreover, Gloria et al. reported the presence of Kiss1 and Kiss1R in dog testis, as well as the presence of Kiss1R in the membrane of spermatozoa recovered from the tail of the epididymis, but not on spermatozoa retrieved from the head and body of the epididymis. The obtained results led the authors to suggest a potential new functional role of the kisspeptin system in the maturation and storage of spermatozoa at epididymal level [58].

It is hypothesized that carbamates may influence the kisspeptin signaling, although the data are scarce. Recently, a study with semicarbazide, a carbamate-related marine pollutant, reported that the exposure of this compound (1, 10 and 100 µg/L for 130 days) by male Japanese flounders (*Paralichthys olivaceus*) resulted in a significant decrease in plasma levels of both testosterone and estrogen due to the downregulation of the expression of steroidogenic enzymes, such as *cyp11a1*, *hsd17b*, *cyp17a1*, and *cyp19a*. Additionally, significantly downregulated transcriptional levels of the G-protein-coupled receptor 54 *(gpr54)*, *gad65*, and *gad67* were found in the brains of male Japanese flounders. As highlighted by the authors, the gpr54, located at the GnRH neurons, binds to kisspeptin to enhance the release of GnRH and GnRH-stimulated gonadotrophins, while L-glutamic acid decarboxylase (GAD), a rate-limiting enzyme for GABA synthesis, can promote the synthesis and secretion of both GnRH and gonadotrophins. Thus, the reduced levels of *gpr54* and *gad* mRNA expression may indicate that semicarbazide is able to inhibit the function of kisspeptin and GABA. Altogether, these results revealed an unconventional mode of action of previously reported anti-estrogenic substances [59]. As aforementioned, kisspeptin is a crucial player in the normal function of the HPT axis, and pollutants like carbamates may lead to its inhibition, thus promoting male fertility disorders. Nevertheless, future studies on this interaction are necessary to validate this assumption.

## 5. Conclusions and Future Perspectives

Carbamates are a class of worldwide used pesticides. The use of pesticides has been intensively studied due the potential harmful effects for the environment, domestic animals, and human beings through environmental exposure and/or the consumption of food products treated with these compounds during their production. One of the most alarming consequences of the chronic exposure to pesticides is infertility. Throughout this review, the reported effects of carbamate exposure in different structures of the male reproductive system were discussed (summarized in Table 1). Although the data are still scarce, the potential role of carbamate pesticides as EDCs is highlighted, thus interfering with natural hormones of the HPT axis and, consequently, affecting the male reproductive system. There is evidence that exposure to carbamates leads to diminished levels of GnRH, LH and/or FSH, thus compromising steroidogenesis and spermatogenesis. Furthermore, several histologic alterations were observed in the testis, which correlated with the diminished male reproductive ability.

The available literature concerning the effects of carbamates on the male reproductive function is, however, still scarce. From the few studies found in the literature, most are in vivo studies using rodent models. These studies, although very informative, have some limitations. The major limitation concerns the chosen concentrations. In some of these studies, animal models are exposed to very high doses of carbamates (and other pesticides), and sometimes the period of exposition is also very large. It must be highlighted that few authors justify the chosen doses, some of which might even be considered unrealistic as they highly exceed the recommended doses for agricultural applications. Thus, further studies using environmentally realistic concentrations of carbamates are needed. In addition, studies at the molecular and epigenetic level are required. Although the current data shed some light on the potential effects of carbamates on the male reproductive function, the molecular mechanisms remain to be disclosed. Pesticides have also been reported to lead to epigenetic modifications, which may persist through several generations. To the best of our knowledge, no studies concerning epigenetic changes due to carbamates exposure have been reported. 

In sum, the potential effects of carbamates exposure on male reproductive health remains to be disclosed. Future studies evaluating the real impact of carbamate pesticides on the male reproductive system and fertility are required, as well as the molecular mechanism of action and potential epigenetic alterations. 

## Figures and Tables

**Table 1:** Effects of in vivo carbamates exposure on male reproductive function.

Reference	Formulation	Dose	Duration of Treatment	Administration	Animal Model	Main Findings
Liu et al. [7]	Carbendazim	0.1, 1, and 10 mg/kg	5 weeks	Oral gavage	ICR mice (*n* = 30 per group)	Decreased sperm concentration and motility Impaired spermatogenesis Decreased estrogen signaling Alterations in histone and DNA methylation
Salem et al. [43]	Carbendazim	100 mg/kg	8 weeks	Oral gavage	Swiss albino rats (*n* = 10 per group)	Decreased sperm concentration, motility, and viability Increased percentage of morphological abnormal sperm Decreased concentrations of serum testosterone, gonadotropins, and inhibin B Increased oxidative stress Alterations on the seminiferous tubules structure
Elsharkawy et al. [44]	Mancozeb	100 mg/kg	12 weeks	Oral gavage (twice per week)	White New Zealand rabbit *(Oryctolagus cuniculus*) (*n* = 9 per group)	Decreased concentrations of serum testosterone and gonadotropins Decreased sperm viability Increased percentage of morphological abnormal sperm Disruption of the germinal epithelium Vacuolization of Leydig cells
Mohanty et al. [45]	Mancozeb	0.14 and 0.28 mg/day	30 days	Oral (mixed with food)	Red Avadavat (*Amandava amandava*) (*n* = 9 per group)	Decreased concentrations of serum testosterone and gonadotropins Impaired gonadal development Altered hypothalamic expression of GnRH Disruption of the HPT axis
Meng et al. [46]	Methomyl	0.2, 2, 20, and 200 μg/L	30 days	Dissolved in water	Nile tilapia (*Oreochromis niloticus*) (*n* = 30 per tank, *n* = 3 per condition)	Altered expression of HPT-related genes in the hypothalamus, pituitary, and testis at 20 and 200 μg/L The effects of 200 μg/L were considered irreversible
Yue et al. [59]	Semicarbazide	1, 10, and 100 μg/L	130 days	Dissolved in water	Japanese flounder (*Paralichthys olivaceus*) (*n* = 6 per group)	Decreased expression of genes involved in steroidogenesis Decreased concentration of serum testosterone and estradiol Disruption of the HPT axis Alterations in the kiss/gpr54 system and GABA synthesis
Guo et al. [47]	Ziram	2 and 4 mg/kg/day	4 weeks	Oral gavage	Sprague–Dawley rats (*n* = 6 per group)	Decreased concentrations of serum testosterone and FSH Decreased Leydig cell number
